# All You Need Is Facebook Friends? Associations between Online and Face-to-Face Friendships and Health

**DOI:** 10.3389/fpsyg.2017.00068

**Published:** 2017-01-30

**Authors:** Maria Luisa Lima, Sibila Marques, Gabriel Muiños, Cristina Camilo

**Affiliations:** Centro de Investigação e Intervenção Social (CIS-IUL), Instituto Universitário de Lisboa (ISCTE-IUL), LisboaPortugal

**Keywords:** health, social isolation, Facebook use, friendship, social capital

## Abstract

Positive social relations are known to have a beneficial impact on health, however, little is known about the links of health with online relationships. In this study, we compare face-to-face and virtual friendships in their association with health. By building on previous results of studies conducted on the well-being of college students, we expect to find stronger associations of face-to-face friendships with health than of those established through Facebook. Furthermore, we expect to test the mediating role of social capital variables in this process. Two large-scale studies conducted in community samples (Study 1 = 350 urban residents; Study 2 = 803 urban and rural residents) showed that the number and quality of face-to-face friendships were directly associated with self-reported health status, however, the same did not occur with Facebook friendships. Moreover, the association of face-to-face friendships with health was totally mediated by bonding (mostly) but also bridging social capital. These results, replicated in both studies, were found controlling for confounding variables such as age, gender, education, living alone, self-esteem, and socioeconomic status. This pattern of results emphasizes the gains of face-to-face over online friendships for individuals’ health status in community samples.

## Introduction

Social networks are now part of our social environment. We find people checking their Facebook account and interacting with their Facebook friends on all types of public transport, in coffee shops or around pools. This new form of social behavior has seen a continuous increase. By June 2015, Facebook, created in 2004, had 1.49 billion monthly active users (Facebook, 2015), i.e., 1/5 of the world population. Although, this is a recognized worldwide phenomenon, the study of the social aspects of this new form of interaction is in its beginnings. Has Facebook changed our idea of friendship? Do Facebook friendships have the same attributes as face-to-face ones? This paper reflects upon these questions and sets out to test, in relation to Facebook friendships, one of the established associations of face-to-face positive social relations: health. It is widely acknowledged that those who have good social ties have fewer illnesses and live longer (e.g., Holt-Lunstad et al., 2015); will this also be the case for Facebook friendships? In two studies conducted with community samples, we compare the relationship of online and face-to-face friendships with health.

## Social Relations and Health

The link between social ties and health has been known for a long time (e.g., Durkheim, 1897/1986; Berkman and Syme, 1979; House et al., 1988; Cohen, 2004), however, recent research and meta-analyses have unsealed dramatic aspects of this association. Loneliness predicts an increased risk of mortality over a 6 year period (Luo et al., 2012) and both objective and subjective measures of social isolation are associated with an increase in around 30% of the likelihood of mortality (Holt-Lunstad et al., 2015). On the contrary, those who have stronger social relationships present 50% more likelihood of survival (Holt-Lunstad et al., 2010) and particular associations with good health have been found in individuals involved in reciprocal and trusting social relationships (Gilbert et al., 2013). In line with this research, the absence of social ties has been conceived as a public health problem, comparable to smoking, alcohol consumption, lack of physical activity and obesity (Holt-Lunstad et al., 2010).

Although, there is a strong body of empirical support for this association, several processes have been advanced as mediators between social ties and health, including biological processes (Cacioppo et al., 2015), emotional processes (e.g., Salovey et al., 2000; Kiecolt-Glaser and Newton, 2001), social support-related processes (Steptoe and Ayers, 2004), and social validation (Jetten et al., 2010). In recent years, social capital, the different value attributed to the social relations as proposed by Putnam (2000), has been used in this context. This author distinguishes two types of social relations with different links to health: bonding and bridging. The first, bonding social capital, is associated with reciprocal relationships amongst similar others, and with the creation of intimate and supportive forms of connectedness that root personal identities; these interactions, common among family members, are characterized by strong social ties, high social support and loyalty (Jensen and Jetten, 2015). The second, bridging social capital, refers to more casual relationships amongst people who are dissimilar and that go across group boundaries; these interactions (for example among neighbors) give access to information outside of the immediate network and build communalities that are important for shared identities (Jetten et al., 2014). So, while bonding refers to strong emotional and close relationships, the weaker ties of bridging are extremely important to social integration and community building. The processes that link these two types of social resources to health are different. For bonding there is a huge psychological literature that illustrates the link of social support to health (e.g., Uchino, 2009), while for bridging there is recent psychosocial evidence for the positive effects of social identification on health (e.g., Haslam et al., 2009; Jetten et al., 2012).

There are several possible theoretical paths to account for these links, and they are summarized elsewhere (e.g., Uchino, 2004, 2009; Haslam et al., 2009). Reciprocal personal relationships are associated with health because they represent a strong form of social control to contain unhealthy behaviors, because they can act as social support and buffer the negative effect of stress on health, because they cause very positive emotions that strengthen the immune system, or because they provide the occasion to share important emotional events with significant others and to give them meaning. In Uchino (2004) perspective, they fight *emotional loneliness* (p. 120). Social integration acts on health through different routes. Participating in community life and interacting with others increases awareness of social norms, provides opportunities for social comparisons (and thus to strengthen self-esteem) and gives access to relevant health information. For Uchino (2004) this type of connection prevents *social loneliness* (p. 120). The relative importance of the two paths to health has not been very much studied. However, a recent meta-analysis of social capital variables and health reports evidence that although the two types of variables are associated with health, effects are stronger for bonding than for bridging (Gilbert et al., 2013), probably due to the importance emotional variables.

Friendship is a voluntary type of social relationship that encompasses intimacy, equality, shared interests, and pleasurable or need-satisfying interactions (Blieszner and Roberto, 2003). Although friendship is a form of social relation and can be seen as social capital, studies on friendship and health are scarce. It is far more common to find work on loneliness and social support that is generalized to friendship. In this paper, we will test the link between friendship characteristics and health, and the mediating role of bridging and bonding social capital.

## Facebook Friendships and Health

Online social networks are undoubtedly one of the main forms of communication in our contemporary societies. Having emerged in 2004, Facebook has become one of the most popular social networks on the Internet, enabling individuals to possess a personal presentation page, to build a network of “friends” and interact with them in various ways such as, for example, by viewing the information on their personal pages and/or posting comments. Given its importance and intensity of use, Facebook is a very rich set of research data that makes it possible to study the nature of online social relationships.

In this study, we are particularly interested in understanding how friendship relations established through Facebook have an impact on the general health of its users. We are also especially interested in exploring the extent to which such effects resemble those observed with networks of face-to-face or oﬄine friends. In an innovative manner, our goal is to explore these effects in a wide community sample. As far as we know, this is the first study to ever study these effects.

Literature on the effects of Facebook friends on health is very recent, and not only shows contradictory evidence but also lacks generalization. The majority of the studies conducted in this domain have only tested university students, thus, reaching different conclusions. Some studies in this area have found that greater use of the Internet has had a negative impact on family communication and given rise to less investment in face-to-face networks of friendships. The HomeNet project (Kraut et al., 1998), which sought precisely to explore the effects of using the internet, found that participants who used the Internet more often reported higher levels of loneliness and a higher number of daily stressful events than people who did not use the Internet so regularly. Subsequent studies (Moody, 2001) also showed that increased use of the Internet (e.g., time spent on the Internet) was associated with a higher degree of emotional loneliness (e.g., absence of intimate relationships), suggesting that the relationships established over the Internet did not meet the social connection needs of individuals and were even capable of inducing depressive states. These results were later replicated by other authors (Caplan, 2007; Ceyhan and Ceyhan, 2008). In general, many of the initial studies on the effects of using Internet seem to point to the fact that its use steals time spent on actual interactions, leading to the isolation of its users and harmful effects on their health and well-being. However, another type of research seems to suggest that this is not always the case. Some studies show that the use of Facebook among university students may be an important trigger for social capital in its several dimensions, and particularly by increasing the bridging dimension. The studies by Ellison et al. (2007) showed that the use of Facebook promoted integration of students in the university campus, their willingness to support the community and to keep “weak ties” with new people in the campus. These effects were especially true for those students with lower self-esteem levels. Following up on these results, Ellison et al. (2011) showed that the number of Facebook friends had no effect neither on bridging nor bonding forms of social capital. However, the number of actual friends (those who are considered to be close friends) did have an effect on both forms of social capital.

Although interesting, the results of these studies still lack generalization. In fact, it is unclear whether these effects of Facebook usage in the specific context of university students would still occur when we consider a varied community sample of different user types. Moreover, it is also still unclear whether friendships established through Facebook would have any added value above oﬄine effects of friendship, when we consider these two types of friendships together.

As far as we know, only one study has explored the relationship between the number of face-to-face friends and Facebook friends and the well-being of the general population. Using a large sample of Canadian respondents in an online survey, Helliwell and Huang (2013) directly compared the effects of the number of oﬄine friendships with the number of Facebook friends on levels of well-being. The results of this study showed that, when considered together, only the face-to-face number of friends had a significant positive effect on well-being. This effect did not appear when considering only Facebook friends. In fact, when considering more sophisticated data analyses (i.e., logged continuous values to express the sizes of the networks), the increase in the number of Facebook friends was associated with a significant decrease in well-being: doubling the number of Facebook friends was equivalent to a 10% decrease in income levels. These more positive effects of face-to-face friendships over Facebook friendships for well-being were similar regardless of marital status, gender or age group. Negative effects of having a higher number of Facebook friends on well-being were particularly higher for middle-aged females in the sample. This suggests that the results obtained with university samples (Ellison et al., 2007) may not be generalizable when we consider a broader variety of Facebook users. However, these results still warrant further exploration. In fact, the sample used in this study was an online non-representative sample, which, once again, makes generalization of these results difficult. Furthermore, this study explores the effects of face-to-face and online friendships on well-being, neglecting other types of fundamental mental and physical outcomes for individuals’ health levels. Finally, this study does not explore the role of possible mediating variables either; and that might help to explain the differential pattern of effects on health of these two different types of friendships.

The present studies aim to overcome these limitations by exploring these processes in community samples both in an oﬄine and online survey. More specifically, this research is also a pioneer in testing the mediating effects that social capital variables – bridging and bonding – may have on these processes.

## Objectives and Overview

This paper compares the association of face-to-face and online friendships with health, and the mediating role of bridging and bonding social capital in this process. Based on previous research, we expect face-to-face friendships to have a positive association with health, and this association will be mediated by bonding social capital (mostly) but also by bridging social capital. Online friendships are expected to have weaker associations with health than face-to-face ones, and to be mostly linked with a reinforcement of face-to-face friendship contacts and bridging social capital.

In order to test these hypotheses, two studies were conducted in the general population. The first study was a telephone survey and the second was an online survey.

## Study 1

This study aimed to compare online and face-to-face friendship associations with health, and to test the mediating role of social capital variables. Studies on Facebook friendships have been mainly conducted with college students. However, given the spread of Facebook to all ages and social strata, it is important to test our hypotheses with a more diverse sample.

### Method

#### Participants

A total of 350 individuals (56% men) accepted participation in this study. The sample had a balanced age distribution: 48.3% of the sample was under 46 years old and the mean age was 46.4 (*SD* = 17.1). Twenty-one point two percent had no completed any school education, 20.3% had completed primary school education, 23.1% had completed secondary education, and 35.4% had an university degree. More than half of the participants (50.6%) were married, 31.1% were single, 12% were divorced, and 6.3% were widowed. Only 21% lived alone. Regarding participants’ employment status, 52% were employed, 20.9% were retired, 15.1% were unemployed, 9.1% were students, and 2.9% were housekeepers. A total of 230 participants (65.7%) had a Facebook account.

Gender, age, and educational level distribution of participants were pre-set to match the characteristics of the populations of Lisbon and Porto, in accordance with the latest census of the Portuguese population (Instituto Nacional de Estatística [INE], 2012).

#### Measures

##### Friendship

Friendship was assessed via two groups of variables: size of friend network and quality of friendships. In each case, the questions focused on both face-to-face and online relationships.

In order to measure the size of the network of face-to-face friends, the items from the Happiness Survey Monitor (Helliwell and Huang, 2013) were used: “Approximately how many friends do you have?.” This question was answered on a scale of 1 *less than 5 friends* to 5 *more than 50 friends*. The question to assess the size of online friend networks was similar (Helliwell and Huang, 2013): “Approximately how many friends do you have on Facebook?” but the response scale was different, ranging from 1 *less than 50* to 8 *over 1000*.

The questions on the quality of relationships used in the European Social Survey were adapted to assess quality of face-to-face and online friendships. The questions were: “How many persons do you have with whom you can discuss intimate and personal matters?” and “How many of these persons are your Facebook friends?.” The response scale varied from 1 (*none)* to 7 (*10 or more)*.

A principle components analysis with an oblique rotation was performed on the four friendship items. Examination of the Kaiser–Meyer Olkin measure of sampling adequacy (*KMO* = 0.511) and of the Bartlett’s test of sphericity (χ^2^_(6)_ = 494.14, *p* < 0.001) suggested that the sample was factorable.

A two-components solution accounted for 82.87% of the variance in the four friendship item scores. Factor 2 aggregated items referring to face-to-face friendship (“Approximately how many friends do you have?” and “How many persons do you have with whom you can discuss intimate and personal matters?”) and factor 1 aggregated the items corresponding to Facebook friendship (“Approximately how many friends do you have on Facebook?” and “How many of the persons with whom you can discuss intimate and personal issues are your Facebook friends?”). Individually, the amount of variance (after rotation) accounted for by factors 1 and 2 was (eigenvalues in parentheses) 55.90% (2.24), and 26.97% (1.08), respectively. In order to determine the internal consistency of the two components, the Pearson correlation was computed for Facebook friendship, *r*_(327)_ = 0.75, *p* ≤ 0.001, and face-to-face friendship, *r*_(341)_ = 0.45, *p* ≤ 0.001. The correlation between the two factors was *r*_(349)_ = 0.34, *p* ≤ 0.001. Structural equation values also show that both face-to-face friends’ observed variables (χ^2^_(30)_ = 60.114; *p* = 0.001) and Facebook friends’ observed variables (χ^2^_(49)_ = 80.770; *p* = 0.003) are related to each other. Factor scores were used as indexes of friendship in the analyses.

##### Health

From a comprehensive health perspective, this questionnaire included indicators of physical health, mental health, and subjective well-being.

In order to assess physical health, in addition to the perceived health item often used in international surveys (e.g., Eriksson et al., 2001 – “How do you rate your health in general” with answers on a 5-point scale, ranging from very good to very bad) the four items of the physical health dimension of the State of Health Questionnaire SF-36 (Ware and Sherbourne, 1992) were included. An example of the questions is: “I seem to get sick a little easier than other people.” These items were answered on a 5-point scale, ranging from 1 *absolutely false* to 5 *absolutely true*. The five items presented an adequate reliability (α = 0.72) and the average of the five items was computed as the indicator of self-reported physical health.

Mental health was assessed via five items from the SF-36 (Ware and Sherbourne, 1992). An example is “How often have you felt very nervous over the last 4 weeks?.” These questions were answered on a 5-point scale, ranging from *never* to *always*. As the level of internal consistency of the items was good (α = 0.80), the indicator of mental health was computed, averaging the responses to these five questions.

The assessment of subjective well-being was performed with two items (*r* = 0.722; *p* < 0.001) answered on an 11-point scale (0–10), which focused on happiness and life satisfaction. These questions are often used for this purpose in surveys such as the European Social Survey (Diener, 2000; Swift et al., 2014).

The level of internal consistency of the three components of health was good (α = 0.81) and only one factor with an eigenvalue greater than one was extracted in an exploratory factor analysis, this factor explained 58.6% of the variance, so items were aggregated in a single index, by computing their average.

##### Social capital

Two variables were included to assess social capital: bonding and bridging. These variables were operationalized using psychological constructs that fit into the definition of the two types of social capital: for bonding, social support, social trust, and (lack of) loneliness; for bridging, social integration, multiple identities, and social interaction.

Bonding social capital was assessed with four reversed items from the USL-4 UCLA loneliness scale (short version; Russell et al., 1980), four items from the short-range version of the social support scale by Haslam et al. (2005; created from the dimensions identified by House, 1981), and one item on social trust: “To what extent do you think you have people you can trust completely?.” The scale on the subjective feeling of loneliness includes items such as “How often do you feel that people around you do not share your interests?.” The answer is given on a scale that ranges from 1 *never* to 5 *almost always*. An example of a social support item is “When you are ill, do you get the help you need?.” Response to these items and to the trust item was given on a scale from 1 *not at all* to 5 *often*. Due to the fact that the level of internal consistency was adequate (α = 0.78), and that one extracted factor was able to explain 37.67% of the variance, the items were aggregated, by computing their average.

Bridging social capital was assessed through an index including items referring to multiple identities, social integration, and general bridging. Three questions assessed the extent to which people belong to multiple social groups, based on items used by Haslam et al. (2008) and Jetten et al. (2010): “I belong to many different groups,” “I participate in several different group activities,” “I have friends from very different groups.” The two items on social integration were the two items of the respective sub-scale of the social well-being questionnaire (Keyes, 2007): “I feel close to the people of the area where I live” and “I am a member of my community.” Finally, two items were adapted from the Bridging Social Capital Scale by Ellison et al. (2007): “Interacting with people makes me discover new things” and “I’m always meeting new people.” The response scale ranged from 1 *strongly disagree* to 5 *strongly agree*. Internal consistency was adequate (α = 0.75), so items were aggregated, by computing their average.

##### Sociodemographic variables

Multiple demographic variables associated with health were measured including gender, age, marital status, education level, and living alone. Furthermore, the one item Scale of Subjective Social Status (Adler et al., 2000; Ostrove et al., 2000) was also included, as it has shown a good level of association with objective measures of social wealth (Operario et al., 2004). Participants were asked to “Think of a ladder with 10 steps representing where people in Portugal stand. The people who are the best off – those who have the most money, the most education, and the most respected jobs are on step 10. Those who are worst off – the people who have the least money, least education, and the least respected jobs or no job are on step 1. Where would you place yourself on this ladder?.” Finally, a one item measure of self-esteem was also included (Robins et al., 2001). Participants were asked to rate their agreement on a 1–5 Likert scale considering the following statement “I have high self-esteem.”

#### Procedure

The research team drew up guidelines and procedural rules regarding characteristics of the sample and of the protocol. The studies included in this paper were approved by ISCTE-IUL Ethical Committee (P04/2016). The study was conducted by telephone, and the fieldwork was assigned to a specialized company. The company procedures followed the ICC/ESOMAR International code on market and social research. The participants were invited to participate in a study on “personal and social relationships.” For those who accepted, the anonymity and confidentially of the answers were guaranteed. Participation was voluntary in all instances and participants were selected from a pre-existing pool, based on a stratified sample, considering age, gender, and education. Individual answers were thus handled anonymously in coding and analyses. After specific consent of the participant, the phone calls were recorded in order to be audited. The questions were asked and answered orally and registered by the interviewer on a pre-prepared database.

### Results

Descriptive analysis revealed strong social ties in our sample. Forty-seven per cent of the participants claimed to have 10 or more friends and 68% referred to having three or more friends with whom they could share intimate issues. Women reported a lower number of friends when compared to men [χ^2^_(5)_ = 18.75; *p* < 0.01] and younger participants reported having a higher number of friends [χ^2^_(25)_ = 47.46; *p* < 0.01] and more close friends [χ^2^_(30)_ = 73.92; *p* < 0.001] than the older participants. However, 16% of the respondents claimed to have less than five friends and around 8% referred having no close friends at all. This percentage was higher among those aged 55 or above; in fact 16% of them mentioned having no one to speak to about intimate issues compared with 3% of those under 55 years old [χ^2^_(30)_ = 73.92; *p* < 0.001].

Sixty-six per cent of our respondents had a Facebook account. Almost half of this group had more than 300 Facebook friends (49%), and 12% had more than 1000 Facebook friends. However, they acknowledged that only a small minority of these were face-to-face friends, and the majority of the participants (64%) had more than three of their close friends as Facebook friends. As was the case for face-to-face friends, younger participants had more Facebook friends than the older ones [χ^2^_(35)_ = 95.07; *p* < 0.001].

Means, standard deviations and bivariate correlations of study variables are presented in **Table 1**. All variables showed significant associations with each other, however, correlation values were weak to moderate, indicating that they measured different constructs.

**Table 1:** Means, standard deviations, and bivariate correlations of Study 1 variables.

	1	2	3	4	5	Mean	*SD*
1. Face-to-face friends	–					3.52	1.34
2. Facebook friends	0.34^∗∗^	–				2.86	2.41
3. Bonding	0.36^∗∗^	0.24^∗∗^	–			4.10	0.60
4. Bridging	0.35^∗∗^	0.33^∗∗^	0.33^∗∗^	–		3.47	0.66
5. Health	0.24^∗∗^	0.16^∗∗^	0.46^∗∗^	0.31^∗∗^	–	4.39	0.62

^∗^*p < 0.05; ^∗∗^*p* < 0.01; ^∗∗∗^*p* < 0.001*.

In order to test the link between friendship variables and health, a hierarchical regression analysis was conducted (**Table 2**), controlling in the first step for some known predictors of health status: age, gender, education, subjective SES, self-esteem, and living alone. These variables explained 29% of the variance in health status. The introduction of Facebook and face-to-face friendship in the regression analyses significantly increased explained variance of health up to 32% (*p* < 0.01). However, this increase was only due to the contribution of traditional forms of friendship, β = 0.15, *p* < 0.01, as online friendship was not associated with health, β = 0.08, *p* = 0.242.

**Table 2:** Study 1: summary of hierarchical regression analysis for variables predicting health (*N* = 350).

	Model 1			Model 2		
	*B*	*SE B*	*β*	*B*	*SE B*	*β*
Gender	0.03	0.06	0.03	0.00	0.06	0.00
Age	-0.01	0.00	-0.18^∗∗^	-0.00	0.00	-0.12
No education	0.20	0.10	0.13^∗^	0.25	0.10	0.16^∗^
Primary education	0.17	0.09	0.11^∗^	0.22	0.09	0.14^∗^
Secondary education	0.17	0.08	0.12^∗^	0.18	0.08	0.12^∗^
SES	0.11	0.02	0.30^∗∗∗^	0.11	0.02	0.29^∗∗∗^
Living alone	-0.09	0.07	-0.06	-0.08	0.07	-0.05
Self-esteem	0.32	0.05	0.35^∗∗∗^	0.31	0.05	0.34^∗∗∗^
Facebook friends	–	–	–	0.05	0.04	0.08
Face-to-face friends	–	–	–	0.10	0.03	0.15^∗∗^
*R*^2^		0.29		0.32		
F for change in *R*^2^		16.01^∗∗∗^		6.03^∗∗^		

^∗^*p < 0.05; ^∗∗^*p* < 0.01; ^∗∗∗^*p* < 0.001*.

**FIGURE 1:**
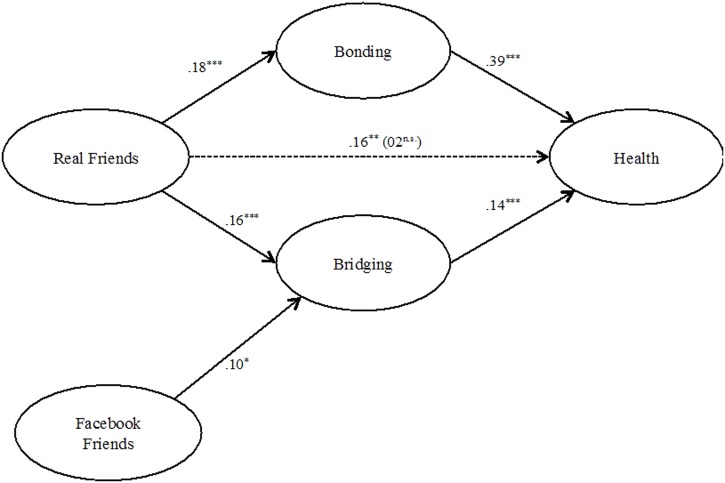
**Model of mediation of bonding and bridging between type of friendship and health**.

To further explore the relationship between friendship and health, we used conditional process modeling to test for mediation, as outlined by Hayes (2013) using the PROCESS macro^1^ (**Figure 1**). More specifically, we tested whether bridging and bonding social capital mediated the relationships among face-to-face friendship and health, as the direct pathway between Facebook friends and health proved not to be significant, β = 0.09, *p* = 0.192. Age, gender, education, subjective SES, self-esteem, and living alone effects were controlled. Missing data were handled with listwise deletion.

The direct pathway between face-to-face friendship and health was totally mediated by bonding and bridging social capital. For the mediation of bonding, we found significant paths between friendship and bonding, β = 0.18, *t* = 5.59, *p* < 0.001, and between bonding and health, β = 0.39, *t* = 7.03, *p* < 0.001. The indirect pathway through bridging was also significant, while the association between friendship and bridging, β = 0.16, *t* = 4.79, *p* < 0.001, and between bridging and health, β = 0.14, *t* = 2.71, *p* < 0.001, were both significant. The direct association between face-to-face friends and health ceased to be significant with the introduction of the mediators, β = 0.02, *t* = 0.51, *p* = 0.613. Comparing the effect sizes, the standardized value (indirect effect) of the mediation path for bonging (0.14) is higher than for bridging (0.06)

Even though the direct pathway between Facebook friends and health proved not to be significant, β = 0.09, *p* = 0.192, we also tested whether bridging and bonding social capital mediated the relationships among Facebook friendship and health (PROCESS; Hayes, 2013). Again age, gender, education, subjective SES, self-esteem, and living alone effects were controlled. Missing data were handled with listwise deletion. We didn’t find a significant regression between Facebook friendship and bonding, β = 0.07, *t* = 1.62, *p* = 0.108, although bonding significantly predicted health, β = 0.35, *t* = 6.92, *p* < 0.001. The association between friendship and bridging, β = 0.10, *t* = 2.24, *p* = 0.026, and between bridging and health, β = 0.10, *t* = 2.13, *p* = 0.034, were both significant. The direct association between Facebook friends and health once the mediators were included wasn’t significant, β = 0.03, *t* = 0.84, *p* = 0.399.

### Discussion

This study shows that online friends have a limited relationship with health, but conversely, that face-to-face friendships present a clear link to well-being. In fact, our community sample reported a high level of social connection, both directly and online, however, only face-to-face friendship significantly predicted health. This association was totally mediated by bonding and bridging social capital, showing that these two paths correspond to important social psychological processes. However, and as predicted, the perception of social support, trust, and the absence of loneliness (psychological processes associated with bonding) were more important in predicting health than multiple identities, sense of community and diversity (the psychological processes associated with bridging). Facebook friendships were linked to bridging social capital (but not to bonding), and this link may also be indirectly connected to health.

These results are relevant given that it is the first time that online and face-to-face friendships are compared in their relationship to general health in a community sample. However, in this study we need to find stronger evidence of the benefits of Facebook friends over face-to-face friends for health.

## Study 2

The goal of this study was to replicate the results obtained in Study 1, with a larger sample, more similar to the Portuguese population, and with recourse to stronger data analysis techniques (structural equation modeling). Moreover, our aim was to further clarify the mediation process, to observe in detail the connection between friendship and health and to compare online and face-to-face friendships, in a sample with high levels of familiarity with the Internet. Thus, an online survey was conducted.

### Method

#### Participants

A total of 803 individuals (50.2% men) accepted participation in this study. Participants were from all over the country, thus including both residents in urban areas (as in Study 1) and in rural areas. Regarding their age, 49.7% of the sample was under 46 years of age and the mean age was 44.1 (*SD* = 15.6). Twelve-point eight percent hadn’t complete any education level, 27% had completed primary education, 29% had completed secondary education, and 31.1% had an university degree; 57.4% of the sample was married, 29.9% was single, 10.9% was divorced, and 1.8% was widowed. Only 10% lived alone. Of all participants, 55.9% were employed, 17.7% were retired, 12.8% were unemployed, 10.3% were students, and 3.2% were housekeepers. A large majority of the participants (89.2%) had a Facebook account. The sample was similar to that of study one, but this time it included residents from all parts of the country. The proportions of participants considering their gender, age, education, and regional distribution were determined to match the characteristics of the Portuguese population, based on the last census (Instituto Nacional de Estatística [INE], 2012).

#### Measures

All the measures included in Study 1 were also included in this online study. The new variables are described below.

##### Friendship

Besides the size of the network of friends, already included in Study 1, in this survey we included questions on the frequency of contact with face-to-face and online friends. In order to assess the frequency of traditional contact with face-to-face friends we asked “How often do you meet socially with friends?.” The response ranged from one *less than once a month* to six *every day*. This item was an adaptation of a question from the European Social Survey (Lima and Novo, 2006; Swift et al., 2014). In order to assess Facebook contacts we asked “How often do you talk to your friends via Facebook?.” In this case the response scale ranged from one *never* to eight *several times a day*. Using a principal component analysis with oblique rotation (*KMO* = 601; Bartlett’s test of sphericity [χ^2^_(6)_ = 436.82, *p* < 0.001], the two items referred to Facebook friends loaded into the same component that explains 47.64% of the variance (eigenvalue = 1.9). The two items about face-to-face friendship loaded in a second component that explains 24.56% of the variance (eigenvalue = 1.0). The correlation between both components was *r*_(803)_ = 0.28, *p* < 0.001. To determine the internal consistency of the two components both the Pearson correlation and the SEM procedure was used. The items for Facebook friendship showed a strong association, *r*_(770)_ = 0.60, *p* ≤ 0.001, χ^2^_(64)_ = 957.634; *p* < 0.001; face-to-face friendship also presented significant associations, *r*_(733)_ = 0.30, *p* ≤ 0.001 and χ^2^_(64)_ = 957.634; *p* < 0.001. Factor scores were used as indexes of friendship in further analyses.

##### Self-esteem

Unlike Study 1, self-esteem was assessed via a shortened version of Rosenberg’s self-esteem scale (Rosenberg, 1965; Pearlin and Schooler, 1978). A six item scale measured self-worth by assessing both positive (five items; e.g., “On the whole, I am satisfied with myself”) and negative (one item; “All in all, I am inclined to feel that I am a failure”) feelings about the self. All items were answered on a 4-point scale ranging from *strongly disagree* to *strongly agree*. As the level of internal consistency of the items was good (α = 0.80), the self-esteem item measure was computed, averaging the items after reverse scoring of the negative ones.

#### Procedure

As in the previous study, the research team drew up guidelines and procedural rules regarding sample and protocol characteristics. The study was conducted via an online platform, and the fieldwork was assigned to a specialized company. The company procedures followed the ICC/ESOMAR International code on market and social research. Participants were invited to take part in a study on “personal and social relationships,” where their answers would be anonymous and confidential. They had to explicitly accept to participate in the study to be directed to the questionnaire. Participation was voluntary in all instances and participants were randomly recruited from a pre-existing pool, composing a stratified national sample, considering age, gender, and education. Answers were automatically registered on a pre-prepared database. Individual answers were handled anonymously in coding and analyses. The data analysis used to test the main hypothesis was structural equation modeling. Specifically, the thresholds for acceptability of the model fit indices were χ^2^/DF < 3; NFI > 0.95; CFI > 0.95; RMSEA < 0.08 with a lower 90% confidence interval < 0.05 and an upper 90% confidence interval < 0.08 (Kenny, 2016, November 22).

### Results

In line with the previous study, this sample revealed strong social ties. Participants claimed to have a considerable number of friends – 59.8% of the sample claimed to have 10 or more friends and 61% referred to having three or more intimate friends. As was the case in the previous sample, women reported a lower number of friends compared to men [χ^2^_(5)_ = 40.09; *p* < 0.01] and younger participants reported a higher number of friends [χ^2^_(25)_ = 38.77; *p* = 0.039] and close friends [χ^2^_(30)_ = 46.40; *p* = 0.028] than the older participants. The other side of the coin reveals that 9.8% of the respondents claimed to have less than five friends and around 8% affirmed having no close friends at all. Regarding older participants, aged 55 years or above, 8.3% referred to not having anyone to speak to about intimate issues and 9.9% indicated having only one intimate friend.

Given that this was an online sample, 89.2% of our respondents had a Facebook account, and a high number of Facebook friends: 41.7% had more than 300 Facebook friends, and 10.7% had over 1000. Nevertheless, only a small minority of these were face-to-face friends: 70.5% of participants had three or less close friends as Facebook friends. As was the case for face-to-face friends, younger participants had more Facebook friends than the older ones [χ^2^_(40)_ = 130.79; *p* < 0.001].

#### Descriptive Statistics

Means, standard deviations, alphas and bivariate correlations of the variables of the study are presented in **Table 3**. Almost all variables showed significant associations with each other, with the exception of Facebook friends, which did not correlate with bonding social capital, mental health, and well-being. All the significant correlations were weak to moderate, indicating that they were measuring different constructs. With the exception of face-to-face friendship (0.443), every alpha score was good. However, due to the fact that this construct was measured by only two significantly correlated items, we considered the lower score as acceptable.

**Table 3:** Means, standard deviations, and bivariate correlations of Study 2 variables.

	1	2	3	4	5	6	7	8	Alpha	Mean	*SD*
1. Face-to-face friends	–								0.437	4.09	1.35
2. Facebook friends	0.28^∗∗^	–							0.737	4.19	2.19
3. Bonding	0.21^∗∗^	0.06	–						0.634	3.67	0.71
4. Bridging	0.36^∗∗^	0.25^∗∗^	0.35^∗∗^	–					0.746	3.40	0.66
5. Health	0.19^∗∗^	0.09^∗∗^	0.59^∗∗^	0.36^∗∗^	–				0.825	4.06	0.68
6. Well-being	0.18^∗∗^	0.05	0.56^∗∗^	0.32^∗∗^	0.83^∗∗^	–			0.941	6.57	1.87
7. Mental health	0.15^∗∗^	0.04	0.49^∗∗^	0.31^∗∗^	0.85^∗∗^	0.58^∗∗^	–		0.784	3.48	0.69
8. Physical health	0.12^∗∗^	0.14^∗∗^	0.35^∗∗^	0.22^∗∗^	0.71^∗∗^	0.34^∗∗^	0.44^∗∗^	–	0.681	3.63	0.61

^∗^*p < 0.05 ^∗∗^*p* < 0.01 ^∗∗∗^*p* < 0.001*.

#### Model 1

**FIGURE 2:**
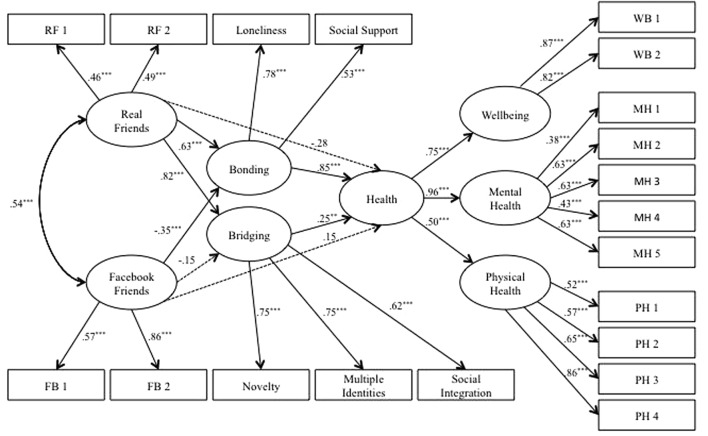
**Structural model of mediation of bonding and bridging between type of friendship and health**.

The first estimated model explored the mediation of bridging and bonding social capital in the association between face-to-face and Facebook friends and health (**Figure 2**). The structural model revealed a good fit to the data, χ^2^ = 446.81, *df* = 153; χ^2^*/DF* = 2.92, *NFI* = 0.95; *CFI* = 0.96; *RMSEA* = 0.05 (90% confidence interval [*CI*] [0.04,0.05]). The results of the model already include a statistical control for the variables of gender, age, living alone, education, self-esteem, SES, and the overlapping between the amount of face-to-face friends and Facebook friends.

The direct paths from face-to-face and Facebook friends to health were not significant. The direct effect of face-to-face friends to health was β = -0.14 (*n.s.*) and the direct effect of Facebook friends to health was β = 0.09 (*n.s.*). However, the indirect path between face-to-face friendship and health was significant. We found significant associations between face-to-face friends, bonding and bridging. The direct effect of face-to-face friends to bonding was β = 0.49, *p ≤* 0.001, and to bridging was β = 0.70, *p ≤* 0.001; and both bonding and bridging were positively and significantly related to health, β = 0.79, *p ≤* 0.001, and β = 0.17, *p ≤* 0.05, respectively. A different result was found for Facebook friends, which had a non-significant association with bridging and a significant but negative association with bonding, β = -0.09, (*n.s.)* and β = -0.34, *p ≤* 0.01, respectively. The full mediation model accounted for 62% of the explained variance of health. Regression weight was β = 0.37 for the association between face-to-face friends and health and *β* = -0.19 for the association between Facebook friends and health.

#### Model 2

A second model was estimated to explore the direct association between Facebook and face-to-face friends and health, nested within the previous mediation. The model was estimated constraining all mediation paths to zero. The constrained nested model fit was χ^2^ = 841.17, *DF* = 160; χ^2^*/DF* = 5.26, *NFI* = 0.90; *CFI* = 0.91; *RMSEA* = 0.07 (90% confidence interval [*CI*] [0.07,0.08]) and it was significantly worse (*p ≤* 0.001) than the fit of the unconstrained model reported in the Model 1 section.

Contrary to Model 1, face-to-face friendship was a significant direct predictor of health, β = 0.24, *p ≤* 0.01. As in the previous model, Facebook friendship was not associated with health, β = -0.13, *(n.s.)*. The whole constrained model accounted for around 5% of the variation of health.

When contrasting Model 2 with Model 1, a significant decrease in the model fit, Δ χ^2^_(6)_ = 377.98, *p ≤* 0.001 may be observed. This statistic indicated that the mediation pathway was, indeed, an important pathway to the model. Therefore, the first model is the best model with an adequate fit to the data.

## General Discussion

According to our hypotheses, these two studies show that face-to-face friendships have a more significant and positive effect on individuals’ health levels than Facebook friendships. Face-to-face friends have an important effect on individuals’ social capital levels, with important and positive effects on health. As expected, and in accordance with the pattern found regarding other variables (e.g., loneliness), we showed that face-to-face friendships may have a significant impact on health both by creating more intimate and supportive links through bonding, and also by giving access to new information and promoting social integration by increasing bridging. More specifically, and in line with previous studies, we showed that the effects of face-to-face friendships on health occurs especially via bonding effects (Beaudoin, 2009; Gilbert et al., 2013).

These results were found controlling for confounding variables that are particularly relevant in a community sample – such as age, SES, living alone, and self-esteem. The usual pattern of results was found (with older, poorer, and low self-esteem participants reporting worse health status) and the effects of friendship appeared over and above the controlled variables. This is an important point since, as the sample was rather diverse, it was possible to replicate well-known patterns of the social determinants of health (Wilkinson and Marmot, 2003). Moreover, our results show an interesting combination of societal and inter-individual factors affecting health, suggesting that positive social relationships can partly compensate for unfavorable material conditions.

These studies also show that when we consider oﬄine face-to-face and online Facebook friendships, only face-to-face friends have positive effects on health. In fact, and similarly to previous studies in this domain (Ellison et al., 2011), although Facebook friends may have positive effects on bridging and health (Study 1), this effect is still smaller than the effects of face-to-face friendships. Furthermore, it is also evident, based on the results of Study 2, that the unique contribution of Facebook friends (independent of face-to-face relationships) may even be detrimental, especially for bonding forms of social capital. Hence, our results seem to be in keeping with previous studies suggesting that the use of the Internet may be associated with high levels of loneliness (Moody, 2001), as it steals time spent on actual interactions and carries harmful effects for the health of its users (Caplan, 2007; Ceyhan and Ceyhan, 2008). These results are also in line with previous results found in community settings exploring the effects on well-being. In fact, and extending the preliminary results by Helliwell and Huang (2013), the present studies, using more robust measurement and analyses, test the role of mediating variables and generalize the results to physical and mental health. In particular, the present results show that Facebook friends actually have a significant negative effect on bonding, thus jeopardizing individuals’ perspectives of developing close and supportive intimate relationships. These results are interesting and show, for the first time, that the results found in university samples (Ellison et al., 2007) do not cover the large community of Facebook users. In fact, it may be that the university context is a specific situation where these types of online connections can be particularly beneficial. However, this does not appear to be the case for the general population of users.

The fact that the negative relationship between Facebook friends and bonding only occurred in Study 2 may be related with the differences between the samples, namely with previous experience in Facebook use. In fact, wheareas in Study 1 65.7% of the respondents referred having a Facebook account, in Study 2 – an online study – 89.2% of the participants reported having such an account. It is possible that the harmful effect of Facebook friends on bonding is stronger for those who use Facebook on a regular basis. However, this is an issue that warrants further exploration in the future.

Literature on the use of social networks, especially by adolescents, has proposed two hypotheses. The first, the social compensation hypothesis (also known as “the poor get richer” hypothesis) states that introverts use the internet to compensate their poor level of interaction. The second one, the social enhancement hypothesis (also known as “the rich get richer” hypothesis) proposes that those more popular increase their social status through online contacts (Zywica and Danowski, 2008; Kuss and Griffiths, 2011). Our results also contribute to this debate, with an adult sample. Facebook and face-to-face friendships were positively related in both studies, and that is consistent with the social enhancement approach. However, the negative association of online friendship with bonding in Study 2 shows support for the alternative compensation hypothesis. As other authors have already shown, probably these strategies are both used but by a different profile of Facebook users (Zywica and Danowski, 2008), and it illustrates the importance of more research on this point. It is also possible that these results are associated with a more or less passive use of Facebook (Kross et al., 2013) and for this reason this variable should be controlled in future studies.

However, despite the important pattern of results found in these studies, they still present some limitations that should be regarded with caution. First of all, it is important to consider that the measures of oﬄine and online friendships are relatively simple. Although this is a common method in these type of studies (Helliwell and Huang, 2013), and we actually measured different aspects of friendship such as closeness (Study 1) and frequency of contact (Study 2), making a generalization of these results more likely, a more complex measure of friendship might still be desirable in future studies. The two item measures used should be improved to for instance, the adoption of the Facebook Use Intensity Scale (Ellison et al., 2007) could possibly be a better way to assess Facebook use. Besides, the more or less passive use of Facebook also seems to be an important variable to be controlled in future research, as suggested by the results of other authors (Kross et al., 2013; Verduyn et al., 2015).

In the same vein, exploration of the possible mediating or moderating effects of different variables affecting Facebook use such as the type of friendship strategies (Ellison et al., 2011) or personal variables such as self-esteem (Kraut et al., 2002; Ellison et al., 2007; Kalpidou et al., 2011; Forest and Wood, 2012) appears to be an important path for the near future. For instance, perhaps Facebook groups exert a very positive influence on a particular type of group such as, for instance, online social support groups (Griffiths et al., 2012). Hence, it is highly important to understand in what type of situations online relationships may be especially beneficial for one’s health.

Finally, it is also important to acknowledge that the correlational nature of these studies does not enable us to completely agree on the direction of these effects. In fact, it may be true that people with lower self-reported health are actually the ones who have both a lower number of face-to-face and online friends. We believe that longitudinal and experimental studies would be of extreme importance in order to allow for a direct test of the direction of these effects.

## Conclusion

We live in a digital era and there is no doubt about that. Nowadays, studying the impacts that the Internet may have on individuals is of paramount importance. This study shows that this “digitalization” of our lives should not replace the value of promoting and keeping oﬄine friendships. Face-to-face friends, with whom we interact in physical settings or through a variety of means, and with whom we can establish caring and close relationships, are fundamental for our health and well-being. Hence, the possibility of living a “second life” in a digital context, where multiple social media networks co-exist, is an interesting possibility, but one that should be regarded with great caution.

## Ethics Statement

The study was approved by the COMISSAO DE ETICA DO ISCTE-IUL. The field work for this study was conducted by a specialized company that followed the The Marketing Research Association’s Code of Marketing Research Standards (ICC/ESOMAR).

## Author Contributions

ML: design of the studies and conception of the paper; state of the art; selection of the instruments; writing. SM: state of the art; data analysis; writing. GM: data analysis; writing. CC: data analysis; writing.

## Conflict of Interest Statement

The authors declare that the research was conducted in the absence of any commercial or financial relationships that could be construed as a potential conflict of interest.

## Footnotes

1 http://www.processmacro.org/index.html
